# CD137 and PD-L1 targeting with immunovirotherapy induces a potent and durable antitumor immune response in glioblastoma models

**DOI:** 10.1136/jitc-2021-002644

**Published:** 2021-07-19

**Authors:** Montserrat Puigdelloses, Marc Garcia-Moure, Sara Labiano, Virginia Laspidea, Marisol Gonzalez-Huarriz, Marta Zalacain, Lucia Marrodan, Naiara Martinez-Velez, Daniel De la Nava, Iker Ausejo, Sandra Hervás-Stubbs, Guillermo Herrador, ZhiHong Chen, Dolores Hambardzumyan, Ana Patino Garcia, Hong Jiang, Candelaria Gomez-Manzano, Juan Fueyo, Jaime Gállego Pérez-Larraya, Marta Alonso

**Affiliations:** 1 Health Research Institute of Navarra (IdiSNA), Pamplona, Spain; 2 Programs in Solid Tumors and Neuroscience, Foundation for the Applied Medical Research, Pamplona, Spain; 3 Department of Neurology, Clínica Universidad de Navarra, Pamplona, Spain; 4 Department of Pediatrics, Clínica Universidad de Navarra, Pamplona, Spain; 5 Program in Immunology and Immunotherapy, Foundation for the Applied Medical Research, Pamplona, Spain; 6 Department of Oncological Sciences, The Tisch Cancer Institut and Department of Neurosurgery, Mount Sinai Icahn School of Medicine, New York, New York, USA; 7 Department of NeuroOncology, The University of Texas MD Anderson Cancer Center, Houston, Texas, USA; 8 Department of Neurosurgery, The University of Texas MD Anderson Cancer Center, Houston, Texas, USA

**Keywords:** oncolytic viruses, brain neoplasms

## Abstract

**Background:**

Glioblastoma (GBM) is a devastating primary brain tumor with a highly immunosuppressive tumor microenvironment, and treatment with oncolytic viruses (OVs) has emerged as a promising strategy for these tumors. Our group constructed a new OV named Delta-24-ACT, which was based on the Delta-24-RGD platform armed with 4-1BB ligand (4-1BBL). In this study, we evaluated the antitumor effect of Delta-24-ACT alone or in combination with an immune checkpoint inhibitor (ICI) in preclinical models of glioma.

**Methods:**

The in vitro effect of Delta-24-ACT was characterized through analyses of its infectivity, replication and cytotoxicity by flow cytometry, immunofluorescence (IF) and MTS assays, respectively. The antitumor effect and therapeutic mechanism were evaluated in vivo using several immunocompetent murine glioma models. The tumor microenvironment was studied by flow cytometry, immunohistochemistry and IF.

**Results:**

Delta-24-ACT was able to infect and exert a cytotoxic effect on murine and human glioma cell lines. Moreover, Delta-24-ACT expressed functional 4-1BBL that was able to costimulate T lymphocytes in vitro and in vivo. Delta-24-ACT elicited a more potent antitumor effect in GBM murine models than Delta-24-RGD, as demonstrated by significant increases in median survival and the percentage of long-term survivors. Furthermore, Delta-24-ACT modulated the tumor microenvironment, which led to lymphocyte infiltration and alteration of their immune phenotype, as characterized by increases in the expression of Programmed Death 1 (PD-1) on T cells and Programmed Death-ligand 1 (PD-L1) on different myeloid cell populations. Because Delta-24-ACT did not induce an immune memory response in long-term survivors, as indicated by rechallenge experiments, we combined Delta-24-ACT with an anti-PD-L1 antibody. In GL261 tumor-bearing mice, this combination showed superior efficacy compared with either monotherapy. Specifically, this combination not only increased the median survival but also generated immune memory, which allowed long-term survival and thus tumor rejection on rechallenge.

**Conclusions:**

In summary, our data demonstrated the efficacy of Delta-24-ACT combined with a PD-L1 inhibitor in murine glioma models. Moreover, the data underscore the potential to combine local immunovirotherapy with ICIs as an effective therapy for poorly infiltrated tumors.

## Introduction

Glioblastoma (GBM) is the most common malignant primary brain tumor in adults, and its prognosis is very poor, with a median overall survival of 14.6 months in newly diagnosed patients and 5-year survival rates lower than 10%.^1–3^ The standard treatment for this tumor consists of surgical resection when possible followed by radiotherapy and temozolomide.^4–7^ Despite paramount efforts to find an effective therapy, GBM remains incurable.

Oncolytic viruses (OVs) have emerged as a feasible treatment for several solid tumors.^8^ OVs are modified viruses that can infect and selectively replicate in tumor cells and thereby exert a cytotoxic effect.^9^ Delta-24-RGD is an oncolytic adenovirus that was modified to specifically kill tumor cells and has demonstrated efficacy in adult glioma models^10^ and pediatric high-grade gliomas, including diffuse intrinsic pontine glioma models.^11 12^ Moreover, the first-in-human clinical trial with Delta-24-RGD (DNX-2401) demonstrated that the virus was safe and effective in a subgroup of patients and led to long-term survivors with a high quality of life.^13^ This trial and others^14^ have uncovered the potential of OVs and the need to further improve their efficacy. Recent approaches suggest that arming OVs with immune costimulatory molecules that can be expressed once tumor cells are infected might induce an effective antitumor immune response.^15–17^

TNFRSF9 (CD137; 4-1BB), which belongs to the tumor necrosis factor superfamily, is a costimulatory receptor present on the surface of activated T cells and natural killer (NK) cells that stimulates T cell proliferation and activation on interaction with its ligands and regulates the activity of immune cells in several physiological and pathological processes.^18 19^ CD137 represents a solid target for cancer immunotherapy because it is highly expressed by CD8+ T cells.^20 21^ Its interaction with CD137L, which is present in different antigen-presenting cells, activates a signaling pathway that promotes CD8+ T cell maintenance and the generation of a memory response. In addition, this signaling pathway plays a pivotal role in the activation of all immune cell populations.^22^ Therefore, we engineered an oncolytic adenovirus (Delta-24-ACT) based on the Delta-24-RGD platform armed with the costimulatory molecule 4-1BB ligand (4-1BBL) to increase the antitumor effect of the adenovirus. Because the composition of the GBM tumor microenvironment is characterized by a large number of myeloid cells but low frequencies of lymphocyte infiltration,^23 24^ we hypothesized that Delta-24-ACT could enhance not only T cell recruitment but also T cell activation. In this study, we aimed to evaluate the antitumor effect of Delta-24-ACT in glioma models. Our results demonstrated that Delta-24-ACT induced a potent antitumor effect in vitro and in vivo by modulating the immunosuppressive glioma microenvironment via the recruitment of immune cell populations. However, this positive outcome was not accompanied by the generation of antiglioma immunological memory because long-term survivors failed to show rejection of tumor rechallenge. Hence, prompted by the increases in PD-1-positive and PD-L1-positive immune cells observed in the tumor microenvironment on Delta-24-ACT, we combined Delta-24-ACT with PD-L1 blockade. This combination prolonged mouse survival compared with that achieved with Delta-24-ACT and PD-L1 monotherapies, mitigated the exhaustion induced by the activation of the immune response and induced an antiglioma memory response in long-term survivors. Importantly, a clinical trial combining Delta-24-RGD and PD-1 blockade in patients with recurrent GBM is ongoing with promising results.^25^ Together, these data underscore the importance of combinatorial approaches for achieving maximal therapeutic efficacy.

## Materials and methods

### Cell lines and culture conditions

The murine glioma cell line GL261 was a kind gift from Dr Safrany (Frederic Joliot-Curie National Research Institute for Radiobiology and Radiohygiene, Budapest, Hungary). GL261-5 (a clone of the GL261 cell line with slow growth)^15^ and CT-2A cells were kind gifts from Dr Fueyo and Dr Gómez-Manzano (UT MD Anderson Cancer Center, Houston, Texas, USA). The GL261-5 cell line was grown in Dulbecco’s modified Eagle’s medium/nutrient mixture F-12 (DMEM/F-12, Gibco), and the GL261 and CT-2A cell lines were grown in DMEM (Gibco, Waltham, Massachusetts, USA). The human glioma cell lines U87-MG and U251-MG were purchased from ATCC and grown in DMEM/F-12 supplemented with 10% Fetal Bovine Serum (FBS) and 1% penicillin-streptomycin. The human cell lines HEK293 and A549 were used for viral replication assays and Delta-24-ACT amplification, respectively. Normal human astrocytes (NHAs) were grown in the AGM Astrocyte Growth Medium BulletKit. All above mentioned cell lines were grown at 37°C in a humidified atmosphere containing 5% CO_2_.

### Delta-24-ACT construction

Delta-24-ACT was constructed by maintaining the following two Delta-24-RGD modifications: (1) the 24-base-pair deletion and the introduction of RGD and (2) the incorporation of the m4-1BBL in the E3 locus after removal of this gene. Briefly, murine 4-1BBL was first cloned into a pCDNA3.1 plasmid using the *Kpn I* and *Xho I* restriction enzymes (New England Biolabs, Ipswich, Massachusetts, USA). Afterward, m4-1BBL flanked with the cytomegalovirus promoter and bovine growth hormone polyadenylation sequences were subcloned into the pAB26-RGD plasmid^26^ at the *Cla I*/*BamH I* sites. Finally, the 4-1BBL expression cassette was introduced into pVK-500C-Δ24, and the Delta-24 plasmid was constructed by homologous recombination with pAB26-m4-1BBL in BJ5183 bacteria. For viral rescue, the obtained plasmid was linearized with *Pac I* and transfected into HEK293 cells with Lipofectamine 2000 (Invitrogen). After confirmation of the genetic modifications by PCR and sequencing, Delta-24-ACT was amplified in A549 cells, purified and stored at −80°C.

### Viral infectivity assay

CT-2A and GL261-5 cells (1.75×10^5^ and 2.5×10^5^, respectively) were seeded in a six-well plate and infected 24 hours later with the AdGFP-Delta-24-RGD adenovirus (Delta-24-RGD with a GFP gene reporter) at multiplicities of infections (MOIs) of 0.1, 1, 10 and 100 for 48 hours. Cells subjected to mock infection (treated with Phosphate-buffered saline (PBS)) were included as a control sample. Green Fluorescent Protein (GFP)-positive cells were analyzed by flow cytometry using a FACSCANTO I flow cytometer (BD Biosciences, Franklin Lakes, New Jersey, USA).

### Viral replication assay

CT-2A and GL261-5 cells (1.75×10^5^ and 2.5×10^5^, respectively) were seeded in a six-well plate. The following day, the cells were infected with Delta-24-ACT at an MOI of 300. NHAs (5×10^4^ cells) were seeded in a six-well plate and infected with wild-type adenovirus^27^ or Delta-24-ACT at an MOI of 10. The cells and supernatants were collected 16 and 72 hours later, respectively. The amount of virus was determined by anti-hexon staining of HEK293 cells as previously described.^28^

### Activity of the transcription factor E2F1

A total of 3×10^5^ cells (24-well plates) were transfected (Fugene 6; E2691; Promega, Madison, Wisconsin, USA) with 250 ng of the plasmid E2F1-Luc expressing a firefly luciferase reporter under the control of an E2F1-responsive promoter.^29^ Additionally, 250 ng of pRL-CMV, which constitutively expresses *Renilla* luciferase (E2261; Promega), was cotransfected as a transfection control. Twenty-four hours later, firefly and *Renilla* luciferase activities were measured using a Dual-Luciferase Reporter Assay System according to the manufacturer’s instructions (E1910; Promega).

### Cell viability assay

CT-2A, NHA (1500 cells/well) and GL261-5 (3000 cells/well) cells were seeded in a 96-well plate. The following day, the cells were infected with Delta-24-ACT at MOIs of 5, 10, 25, 50 and 100, and 5 days after viral infection, the cell viability was assessed using the Cell-Titer 96 One Solution Aqueous Proliferation Kit (Promega). The absorbance was measured using a SPECTROstar Nano reader (BMG Labtech, Ortenberg, Alemania, USA). The data are presented as percentages (means±SDs of three independent experiments) of viable cells after infection with Delta-24-ACT at the indicated MOIs relative to the values found for mock-treated cells (control, equal to 100%). The IC_50_ value was defined as the median-effect dose (dose affecting 50% of the cells). The dose–response curves were analyzed using GraphPad Prism software.

### Crystal violet staining

Human U87-MG cells or NHAs were seeded (3.2×10^4^ cells/well) in 24-well plates and allowed to grow for 20 hours at 37°C. The cells were then infected with Delta-24-ACT at the indicated MOIs and cultured at 37°C for 48 hours. Afterward, the cell monolayers were washed twice with PBS, fixed and stained with 0.1% crystal violet in 20% ethanol. Excess dye was removed by several rinses with water. The cell viability was assessed by resuspending the cells in 10% acetic acid for 5 min. The absorbance of 50 µL per well was measured at 570 nm using a SPECTROstar Nano reader (BMG Labtech).

### Characterization of viral proteins by western blot analysis

CT-2A and GL261-5 cells (1.75×10^5^ and 2.5×10^5^, respectively) were seeded in a six-well plate, and 24 hours later, the cells were mock infected or infected with Delta-24-ACT at MOIs of 25, 50, 100 and 300. The cells were collected at 16 and 48 hours after viral infection. Protein extracts were prepared from the cells by incubation with 100 µL of lysis buffer (PBS+0.1% Triton and a protease inhibitor cocktail) for 30 min on ice. The samples were then centrifuged at 10,000 rpm and 4°C for 15 min, and the supernatant was collected. All protein extracts were quantified using a colorimetric assay (Protein Assay Dye Reagent Concentrate, Bio-Rad, Hercules, CA, USA) following the manufacturer’s instructions and measured using a SPECTROstar Nano reader (BMG Labtech). Thirty micrograms of protein was loaded on and separated in a 10% SDS-polyacrylamide gel under denaturing conditions. Afterward, the proteins were transferred to a nitrocellulose membrane and incubated with the following antibodies: anti-E1A (1:1000, Sc-430 Santa Cruz Biotechnology, Santa Cruz, CA, USA), anti-Fiber (1:1000, NB600-541 Novus Biologicals, Englewood, CO, USA), anti-Grb2 (1:1000, 610112 BD) and anti-4-1BBL (1:1000, AF1246 R&D Systems). The protein bands were detected by enhanced chemiluminescence using a ChemiDoc MP imaging system (Bio-Rad).

### Flow cytometry

Excised tumors were mechanically dissociated using a scalpel, incubated with collagenase IV/DNase (17018-029 Gibco/11284932001 Roche) with rotation for 15 min and then incubated twice for 10 min at 37°C. The solution was filtered through a 70-μm cell strainer (Thermo Fisher Scientific, Waltham, Massachusetts, USA), and after the addition of a 30% Percoll solution (17-0891-01 GE Healthcare, Chicago, IL, USA), tumor cells were isolated by centrifugation at 800×g and 4°C for 15 min. Single-cell suspensions were then stained for flow cytometry. The fluorochrome-tagged monoclonal antibodies (mAbs) used in this assay are listed in table 1. For nuclear staining, the cells were fixed and permeabilized using BD Cytofix/Cytoperm Plus (555028 BD Biosciences) and then stained according to the manufacturer’s instructions.

**Table 1:** Antibodies used for flow cytometry analysis

Antibody	Dye	Species	Dilution	Company	Reference
CD3 Clone 17A2	FITC	Mouse	1:200	BioLegend	100203
CD4 Clone RM4-5	FITC	Mouse	1:800	BioLegend	100510
CD4 Clone GK1.5	BUV496	Mouse	1:200	BioLegend	612952
CD8a Clone 53-6.7	BV510	Mouse	1:400/100	BioLegend	100752
CD8a Clone KT-15	BV421	Mouse	1:200	Bio-Rad	MCA609PBT
CD11b Clone M1/70	BV510/BUV395	Mouse	1:400/200	BioLegend	101263/563553
CD11c Clone N418	PECy7	Mouse	1:160	BioLegend	117318
CD19 Clone 6D5	BV421	Mouse	1:400	BioLegend	115538
CD25 Clone PC61	PerCP/Cy5.5	Mouse	1:100	BioLegend	102030
CD40 Clone 3/23	PE	Mouse	1:200	BioLegend	124609
CD44 Clone IM7	AF594	Mouse	1:200	BioLegend	103054
CD45 Clone 30-F11	APC/AF750	Mouse	1:1000/200	BioLegend	103154/103128
CD86 Clone GL-1	PerCP/Cy5.5	Mouse	1:200	BioLegend	105027
CD86 Clone GL-1	BV510	Mouse	1:1000	BioLegend	105039
CD137 Clone17B5	PE	Mouse	1:200	BioLegend	106105
CD137L Clone TKS-1	PE	Mouse	1:200	BioLegend	107105
F4/80 Clone BM8	APC	Mouse	1:200	BioLegend	123116
FOXP3 Clone 3G3	PECy7	Mouse	1:160	Abcam	ab210232
FOXP3 Clone FJK-16S	PE-eFluor610	Mouse	1:100	eBioscience	615773-80
GITR Clone DTA-1	PerCP/Cy5.5	Mouse	1:200	BioLegend	126316
Granzyme B Clone NGZB	PeCy7	Mouse	1:80	BioLegend	25-8898-80
H-2K^b^/M8	APC	Mouse	1:200	Creative Biolabs	MHC-LC521
IA/IE Clone M5/114.15.2	BV650	Mouse	1:300	BioLegend	107641
Ly6C Clone HK1.4	FITC	Mouse	1:400	BioLegend	128006
Ly6G Clone 1A8	PerCP/Cy5.5	Mouse	1:400	BioLegend	127616
NK1.1 Clone PK136	APC/BV605	Mouse	1:20/100	BioLegend	108710/108739
PD-1 Clone 29F.1A12	BV421	Mouse	1:160/200	BioLegend	135218
PD-L1 Clone 10F.9G2	BV421	Mouse	1:80/200	BioLegend	124315
TCRb Clone H57-597	BV785	Mouse	1:100	BioLegend	109249

For in vitro experiments, murine glioma cells were infected with Delta-24-ACT (MOI of 100) for 48 hours. The marker PD-L1 was analyzed by flow cytometry.

For M8 tetramer analysis, tumor-infiltrating lymphocytes (TILs) were stained with H-2K^b^/M8 (MuLV p15E_604-611_)-tetramer and then with fluorochrome-conjugated mAbs against CD45, CD8, CD4 and TCRb. Detailed antibody information is provided in table 1. The cell viability was assessed using PromoFluor 840 (1:10,000, PK-PF840-3-01 PromoCell) for 15 min at 4°C prior to antibody incubation. Acquisition was performed using a FACSCanto II (BD Biosciences) and a CytoFLEX LX (Beckman Coulter, Brea, CA, USA) flow cytometer.

### RNA extraction and real-time PCR

For in vivo 4-1BBL analysis, tumors were disaggregated with collagenase IV/DNase (17018-029 Gibco/11284932001 Roche). Total RNA was extracted from isolated cells using TRIzol according to the manufacturer’s instructions (Life Technologies, Carlsbad, CA, USA). RNA samples were quantified using a Nanodrop 1000 spectrophotometer (Thermo Fisher Scientific) and stored at −80°C. One microgram of RNA was reverse transcribed using a high-capacity cDNA reverse transcription kit (Applied Biosystems, Thermo Fisher, Bedford, MA, USA). Afterward, cDNA was amplified using SYBR-Green Master Mix (Applied Biosystems). The gene-specific assay was murine 4-1BBL (ACT and endogenous (end)). GAPDH was used as the housekeeping control gene, and all samples were run in triplicate. The sequences of the primers are listed in table 2. Real-time PCR was monitored using an ABI 7700 sequence detection system (Applied Biosystems). The fold changes in the expression of the genes of interest were calculated as the mean values calculated using the 2^–ΔΔCT^ method.

**Table 2:** Primers used for quantitative real-time PCR

Antibody	Species	Dilution	Company	Reference
CD3	Rabbit	1:150	Abcam	Ab5690
CD31	Rat	1:50	DiaNova	DIA-310
PD-L1	Rabbit	1:100	Cell signaling	64988

### IFN-gamma ELISA

CT-2A cells were infected with Delta-24-ACT at an MOI of 100, and 4 hours later, the cells were incubated with recombinant murine IFN-γ (100 IU/mL) for 48 hours. Splenocytes were isolated and cocultured with mock-infected or infected CT-2A cells in a 96-well flat-bottomed plate for 72 hours (ratio 40:1). The supernatant of the wells was collected and analyzed using the mouse IFN-gamma DuoSet ELISA kit (DY485, R&D Systems) following the manufacturer’s instructions. To assess the activation of T cells after viral infection, splenocytes from B6.CgThy1a-Tg(TcraTcrb)8Rest/J (PMEL) mice were isolated and seeded (4×10^5^) in a 96-well flat-bottomed plate coated with an anti-CD3 antibody (clone 145-2 C11, 100314 BioLegend) for 24 hours. CT-2A cells (1×10^4^) were mock infected (control) or infected with either Delta-24-RGD or Delta-24-ACT (MOI of 100). Forty-eight hours later, the cells were incubated with hgp100 (25-33) (1 ng/mL) (RP20344 GenScript) for 2 hours. Splenocytes and cells were cocultured for 3 days. The supernatant was collected after 24 and 48 hours of coculture and analyzed using the mouse IFN-gamma DuoSet ELISA kit (DY485 R&D Systems) following the manufacturer’s instructions.

### IFN-gamma ELISPOT

CT-2A cells were infected with Delta-24-ACT at an MOI of 100. Four hours after cell infection, mock-infected or infected CT-2A cells were incubated with murine recombinant IFN-gamma (100 IU/mL). Forty-eight hours later, splenocytes were isolated from mice and cocultured with mock-infected or infected CT-2A cells (ratio of 10:1) for 24 hours in a 96-well plate. A mouse IFN-γ ELISPOT set (551083 BD) was used according to the manufacturer’s instructions, and the results were measured using an IMMUNOSPOT S6 Analyzer (Macro, IMMUNOSPOT).

### Animal studies

All animal experimental protocols were reviewed and approved by the Committee of Bioethics of the Government of Navarra and the Institutional Animal Care Department (protocol numbers: 090-18 and 097-16). Four to six weeks old C57BL/6 and athymic nude (nu/nu) mice (Envigo Laboratories) were used in this study. The mice were maintained at The Center for Applied Medical Research under specific pathogen-free conditions and fed standard laboratory chow. GL261 and GL261-5 (50,000 cells per mouse in 3 µL of free medium) or CT-2A (5000 cells per mouse in 2 µL of free medium) cells were implanted in the supratentorial region of the mice using a guide-screw system described previously.^30^ Three injections of Delta-24-ACT (1×10^8^ PFUs/mouse, 3 µL) or control (PBS, 3 µL) were administered. In the experiments comparing Delta-24-ACT and Delta-24-RGD, both viruses (1×10^8^ PFUs/mouse, 3 µL) were inoculated once. To evaluate the combination of Delta-24-ACT with PD-L1 blockade, GL261 (50,000 cells per mouse in 3 µL of free medium) cells were implanted as described above. Three intratumoral inoculations of Delta-24-ACT (1×10^8^ PFUs/mouse, 3 µL) were administered on days 5, 7 and 9 after cell implantation. The PD-L1 antibody clone 10F.9G2 (Bioxcell, #BE0101) was administered intraperitoneally on days 7, 10, 13 and 16 after cell implantation. Mice whose survival was three times the median of that of the control mice were considered long-term survivors. For the analysis of 4-1BBL in vivo, 50,000 CT-2A cells were inoculated into the intracranial model. Delta-24-ACT (1×10^8^ PFUs/mouse) was inoculated intratumorally, and 48 hours later, the mice were sacrificed. For subcutaneous models, 500,000 CT-2A or 3×10^6^ U87-MG cells were inoculated. The tumor size was measured using a caliper. Delta-24-ACT or Delta-24-RGD (1×10^8^ PFUs/10 µL) was administered once the tumor volume reached 50 mm^3^. Forty-eight hours later, the mice were sacrificed, and the tumors were removed for RNA analysis. A control group treated with PBS was included. For the mechanistic studies using the combination of Delta-24-ACT with PD-L1 blockade, Delta-24-ACT (1×10^8^ PFUs/mouse, 3 µL) was administered intratumorally once on day 5 after cell inoculation, and PD-L1 antibody was administered twice on days 7 and 9 after cell inoculation.

### Immunohistochemistry (IHC) and immunofluorescence (IF)

The brains were embedded in paraffin blocks, and 3 µm tissue slides were stained with appropriate antibodies (table 3). The slides were visualized using 3,3-diaminobenzidine (DAB, K346889-26 Dako) and counterstained with hematoxylin (HX85602653 MERCK). The preparations were observed under a confocal microscope (0114107 Nikon Y-THS) and scanned using an Aperio C52 image capture device (Leica Microsystems) and Aperio ImageScope 12.1.0 software (Leica Microsystems).

**Table 3:** Antibodies used for immunohistochemistry and immunofluorescence

Gene	Species	Forward	Reverse
*4-1BBL ACT*	Mouse	5′ CTTGTGAAACCCGACAACCC 3′	5′ CAGCGAGCTCTAGCATTTAGGT 3′
*4-1BBL end*	Mouse	5′ AGTGTGGGTCTGAGGGCTTA 3′	5′ AGCAGCTTGAGGACTTAGCAA 3′
*GAPDH*	Mouse	5′ GGGAAATTCAACGGCACAGT 3′	5′ AGATGGTGATGGGCTTCCC 3′
*GAPDH*	Human	5′ AGCCACATCGCTCAGACAC 3′	5′ GCCCAATACGACCAAATCC 3′

### Hepatotoxicity studies

Different biochemical parameters in the murine serum were analyzed. Blood was collected from mice treated three times with Delta-24-ACT (1×10^8^ PFUs/mouse, 3 µL) or PBS administered intratumorally 10 days after viral inoculation using the CT-2A model. Serum was obtained after centrifugation of the blood at 10,000 rpm for 10 min, and the serum samples were maintained at −80°C until hepatotoxicity analyses were performed. For the evaluation of hepatotoxicity, the levels of albumin, alkaline phosphatase (ALP), alanine transaminase (ALT), aspartate transaminase (AST) and total bilirubin (BILT3) were analyzed using a Cobas c 311 analyzer.

### Statistical analysis

The data are presented as the means±SD from three biological replicates and were compared using Student’s t-test. Multiple comparisons were made by one-way ANOVA and the post hoc Bonferroni test. P value <0.05 (*p<0.5, **p<0.01, ***p<0.001 and ****p<0.0001) was considered significant. The survival rate was evaluated using Kaplan-Meier plots, and statistical analyses were performed with the log-rank test. The program used for the statistical analyses was GraphPad Prism software (GraphPad Software).

## Results

### Delta-24-ACT exhibits oncolytic capacity in glioma cells

**Figure 1:**
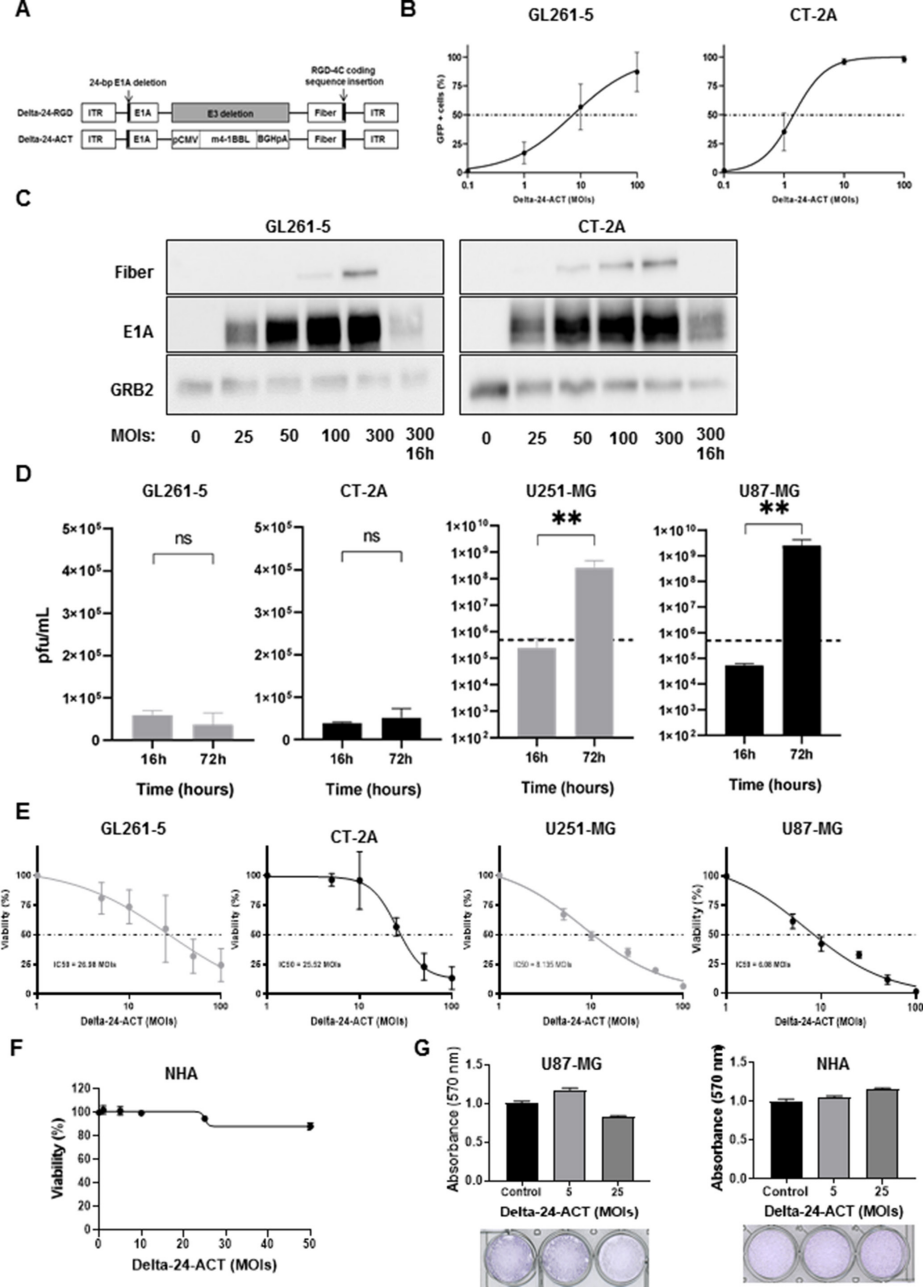
Characterization of Delta-24-ACT in glioma cell lines. (A) Schematic representation of engineered Delta-24-ACT. (B) Infectivity of Delta-24-ACT in GL261-5 (left panel) and CT-2A (right panel) cells measured by flow cytometry as the percentage of GFP+ cells at 48 hours after infection with Delta-24-RGD-GFP at MOIs ranging from 0.1 to 100 PFUs/cell. The values represent the mean percentages of GFP+ cells ±SDs (n=3). (C) Assessment of viral protein expression (Fiber and E1A) in GL261-5 and CT-2A cells by western blot analysis. Cells were infected with Delta-24-ACT at the indicated MOIs, and 48 hours later, whole-cell lysates were collected. Samples were also collected at 16 hours postinfection with the highest dose (MOI of 300) as a control. Grb2 was used as a protein-loading control. (D) Replication and quantification of Delta-24-ACT in murine CT-2A and GL261-5 cells and human U251-MG and U87-MG cells that were infected at MOIs of 300 and 10, respectively. Delta-24-ACT replication was determined 16 and 72 hours postinfection. The dashed lines indicate the total initial viral input, and the results are expressed as the mean viral titers±SDs (n=3, one-tailed Mann-Whitney test). Oncolytic effect of Delta-24-ACT on murine and human glioma cells (E) and NHAs (F). To quantify the oncolytic effect of Delta-24-ACT on murine CT-2A and GL261-5 cells and human U251-MG, U87-MG and NHA cells, the cells were infected at the indicated MOIs, and 5 days later, their viability was evaluated by MTS assays. The values indicate the percentages of viable cells in infected cultures compared with those in noninfected cultures (means±SDs, n=3). (G) Oncolytic effect of Delta-24-ACT on U87-MG cells and NHAs. U87-MG cells and NHAs were infected at the indicated MOIs, and 3 days later, their viability was measured by crystal violet staining. MOI, multiplicities of infection; NHA, normal human astrocyte.

We generated Delta-24-ACT, which is an oncolytic adenovirus based on the Delta-24-RGD platform (figure 1A) that encodes 4-1BBL in the E3 locus. To test whether Delta-24-ACT is able to infect murine glioma cell lines, the GL261-5 and CT-2A cell lines were treated with an Ad5-GFP-RGD virus that expresses GFP at different MOIs ranging from 0.1 to 100, and the infection was quantified by flow cytometry. We observed that the virus could infect both cell lines at low MOIs, and CT-2A cells were more susceptible than GL261-5 cells: almost 100% of the CT-2A cells were positively infected at an MOI of 10, and a lower positive percentage was obtained with GL261-5 cells (figure 1B). In addition, we assessed the expression of two important viral proteins, E1A and Fiber, in infected GL261-5 and CT-2A cells. The E1A protein was expressed in a dose-dependent manner in both cell lines (figure 1C and online supplemental figure 1A). As expected, expression of the early protein E1A but not Fiber, a late viral protein, was observed in both cell lines at 16 hours. Fiber protein expression was also detected at later times, although this expression was more attenuated in both cell lines (figure 1C). Because replication is crucial for the therapeutic effect of OVs and adenovirus 5 replication is hampered in murine cell lines,^31^ we next evaluated the replication potential of Delta-24-ACT in human and murine glioma cell lines. As expected, Delta-24-ACT was unable to replicate in either of the murine cell lines tested. However, the virus efficiently replicated in the U251-MG and U87-MG human glioma cell lines (figure 1D). These data support the potential of Delta-24-ACT as a therapeutic agent. Subsequently, we evaluated the cytotoxic effect of Delta-24-ACT on the different glioma cell lines at 5 days postinfection. Delta-24-ACT exerted a robust cytotoxic effect on all cell lines tested, and the human cell lines were more sensitive to the virus. GL261-5 and CT-2A cells showed IC_50_ values of 29.79±1.26 PFUs/cell and 25.52±1.08 PFUs/cell, respectively (figure 1E). The observed cytotoxic effect is due to the capacity of E1A protein to induce cell death (review in Berk^32^). Furthermore, lower IC_50_ values were obtained with U251-MG and U87-MG cells (8.36±2.3 PFUs/cell and 6.08±1.1 PFUs/cell, respectively; figure 1E). To evaluate the therapeutic index of Delta-24-ACT, we quantified its ability to induce cytotoxicity in NHAs. MTS assays showed that NHAs were resistant to the effect of the virus (figure 1F). Comparison of the Delta-24-ACT cytopathic effect in U87-MG and NHAs again showed that the normal cells were resistant to the virus effect (figure 1G). As shown previously, E2F promoter activity in NHAs was significantly lower than that in glioma cell lines (online supplemental figure 1B). Supporting this notion, Delta-24-ACT was unable to replicate in NHAs compared with wild-type adenovirus (online supplemental figure 1C). In summary, Delta-24-ACT is able to infect cells, retain its replication potential and exert a potent antiglioma effect in vitro while maintaining a good therapeutic index.

10.1136/jitc-2021-002644.supp1 Supplementary data

10.1136/jitc-2021-002644.supp2 Supplementary data

### Delta-24-ACT infection in glioma cells efficiently activates CD8+ lymphocytes

**Figure 2:**
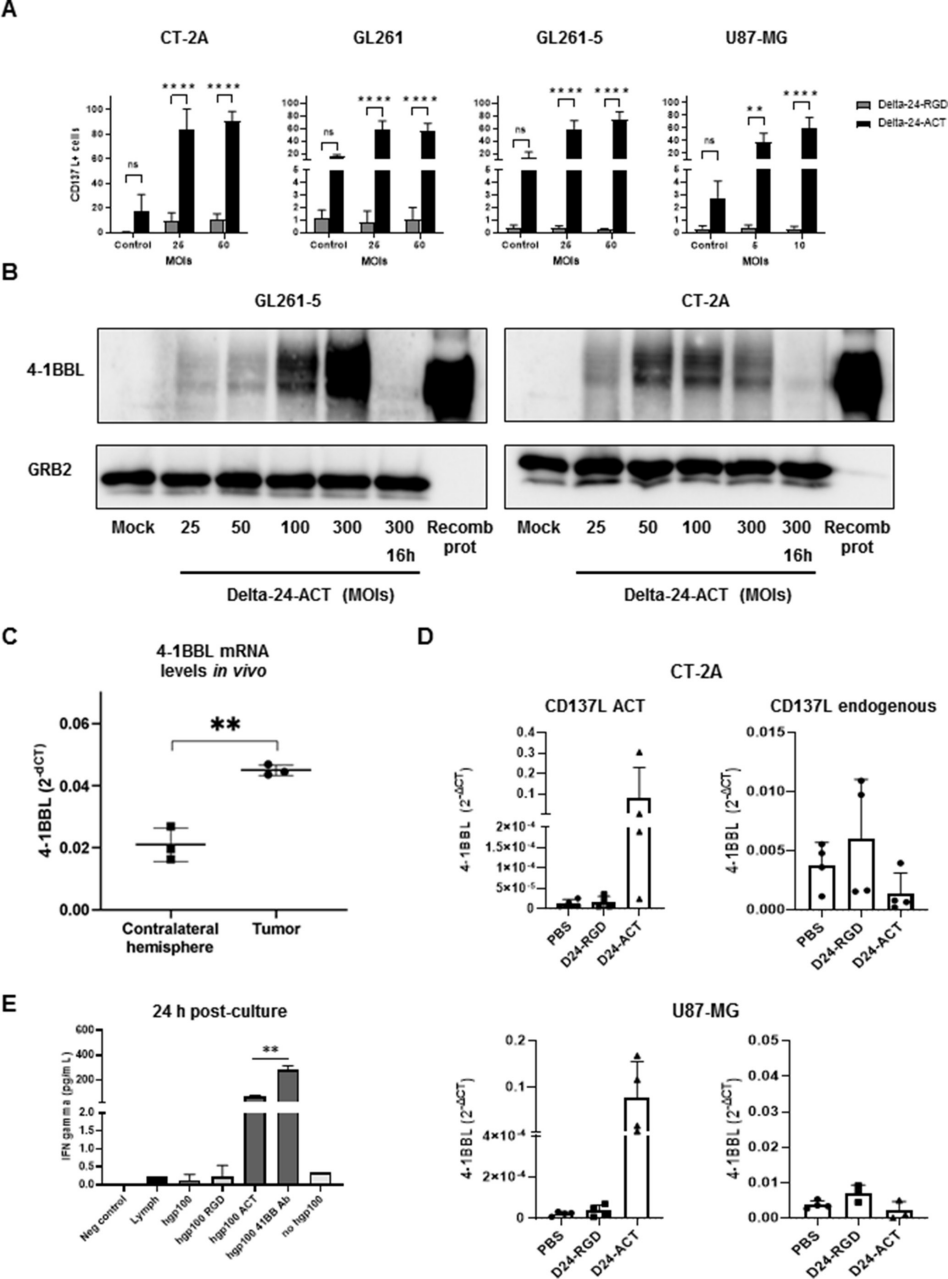
Delta-24-ACT can be expressed in glioma cells and induces T cell activation. (A) 4-1BBL protein expression in CT-2A, GL261, GL261-5 and U87-MG cells infected with Delta-24-ACT and Delta-24-RGD at the indicated MOIs analyzed by flow cytometry (one-way ANOVA). (B) 4-1BBL protein expression in GL261-5 and CT-2A cells infected with Delta-24-ACT at the indicated MOIs analyzed by western blot analysis. A representative image is shown. (C) In vivo evaluation of 4-1BBL mRNA expression. The mRNA expression of 4-1BBL in mice bearing CT-2A tumors 3 days after treatment with Delta-24-ACT was analyzed by real-time PCR. The contralateral hemisphere was used as the negative control (Student’s t-test). (D) In vivo assessment of 4-1BBL mRNA expression in mice bearing CT-2A and U87-MG subcutaneously and treated with either Delta-24-RGD or Delta-24-ACT for 48 hours. Mice treated with PBS were used as controls. (E) IFN-gamma production by lymphocytes after Delta-24-ACT infection. CD8+ T cells from PMEL mice were cocultured with CT-2A cells infected with Delta-24-RGD or Delta-4-ACT at an MOI of 100. The secretion of IFN-gamma in the collected supernatants was quantified by ELISA. MOI, multiplicities of infection.

Because Delta-24-ACT was designed to express the costimulatory ligand 4-1BBL, we subsequently assessed the expression and functionality of this ligand within the virus. Murine and human glioma cells infected with Delta-24-ACT displayed significantly higher 4-1BBL protein expression on the membrane compared with that found in the control cells, as shown by flow cytometry (figure 2A and online supplemental figure 1D). We confirmed the expression of 4-1BBL in murine and human glioma cell lines by western blot analysis (figure 2B and online supplemental figure 1E). Interestingly, we observed a difference in the pattern of expression between the human and murine cell lines, which likely reflected the different posttranslational changes that the protein undergoes in murine and human cell lines (online supplemental figure 1B). We then administered a single viral injection to mice bearing orthotopic CT-2A cells and analyzed the brains 3 days later. Importantly, we detected the expression of 4-1BBL in the treated mouse tumors in comparison to that in normal tissue from the contralateral hemisphere (figure 2C). Additionally, mice bearing CT-2A or U87-MG cells were treated with PBS, Delta-24-RGD and Delta-24-ACT, and 2 days later, the expression of endogenous or viral 4-1BBL was quantified by qPCR. We observed a significant increase in the expression of viral 4-1BBL in CT-2A and U87-MG tumors, whereas the endogenous ligand remained unaltered (figure 2D). To assess the capability of 4-1BBL to trigger lymphocyte activation in the context of viral infection, we cocultured mouse hgp100-specific CD8+ T cells with CT-2A cells pulsed with human hgp100 and previously infected with Delta-24-ACT. Delta-24-ACT efficiently activated these lymphocytes, as shown by IFN-gamma production after 24 and 48 hours of coculture. The secretion of IFN-gamma from lymphocytes cocultured with Delta-24-ACT-infected cells was significantly higher than that from lymphocytes cocultured with Delta-24-RGD-infected cells at both 24 and 48 hours. Unpulsed CT-2A cells and an anti-4-1BB antibody were used as negative and positive controls, respectively (figure 2E). These data underscore the functionality of 4-1BBL when expressed via the Delta-24-ACT virus.

### Delta-24-ACT induces an antiglioma effect *in vivo*

**Figure 3:**
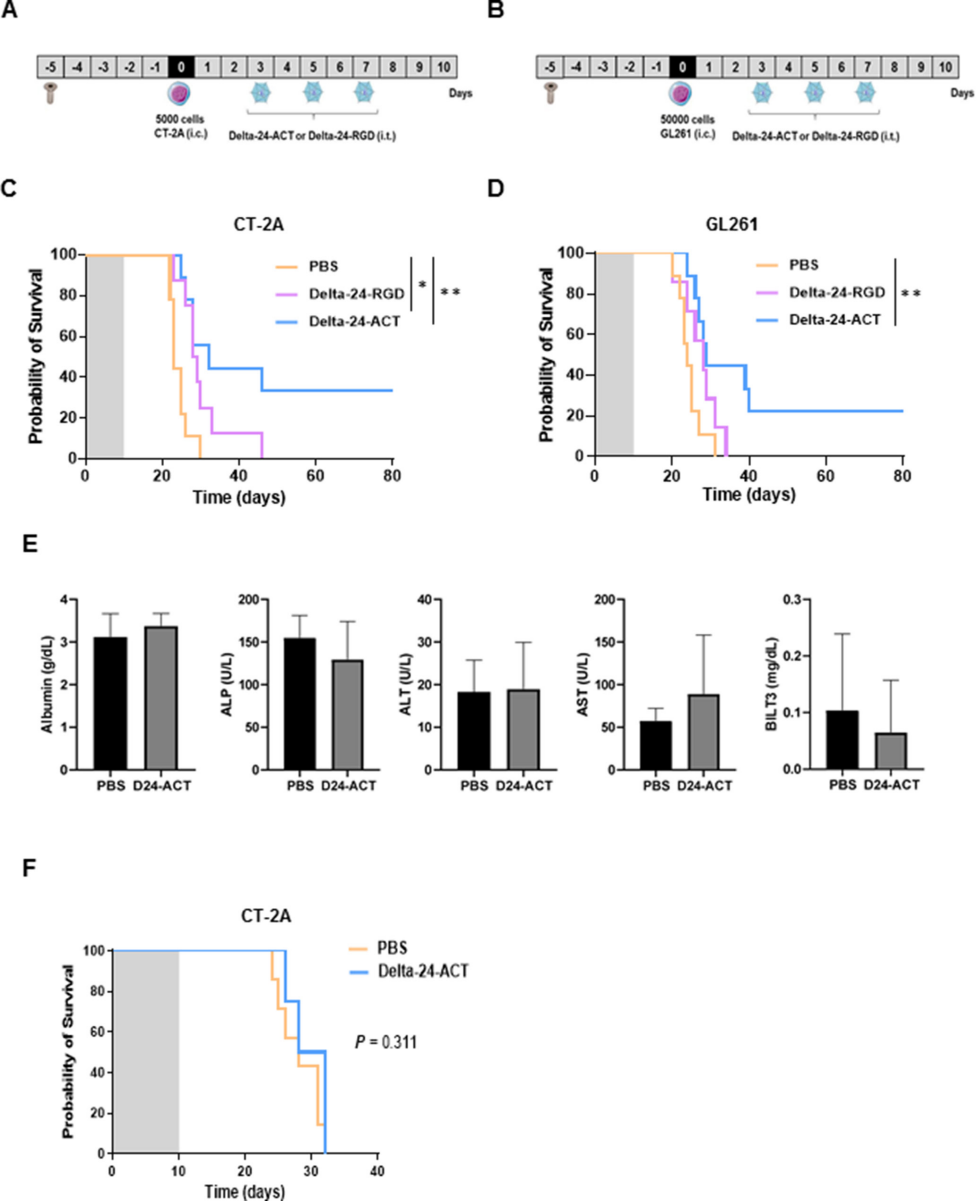
Comparison of the in vivo antitumor effects exerted by Delta-24-RGD and Delta-24-ACT in murine glioma models. Schedule of survival experiments with CT-2A (A) and GL261 (B) tumor models. Survival of mice bearing CT-2A tumors (C) or GL261 tumors (D) treated with Delta-24-ACT or Delta-24-RGD (1×10^8^ PFUs/mouse, 3 µL, n=10). A control group of mice treated with PBS was included in every survival experiment. (E) Biochemical studies performed with the serum of mice bearing CT-2A tumors (n=5) treated with mock infection or Delta-24-ACT analyzed by a Cobas 311 analyzer. (F) Rechallenge experiment involving the inoculation of naïve mice (n=5) or long-term survivors with CT-2A cells. All survival experiment results are shown as Kaplan-Meier survival curves (log-rank test). The shaded area represents a 10-day interval from the time of cell implantation.

We then evaluated the antitumor effect of Delta-24-ACT in vivo. Mice bearing intracranial CT-2A or GL261-5 cells were treated with Delta-24-ACT or a control (online supplemental figure 2A, B). Treatment with Delta-24-ACT resulted in an increase in median survival (PBS=27 days vs Delta-24-ACT=41.5 days; p<0.0001) and led to 42% long-term survival in mice bearing CT-2A tumors (online supplemental figure 2C). The treatment of mice bearing GL261 cells with Delta-24-ACT also led to an increase in median survival (PBS=35 days vs Delta-24-ACT=39 days; p=0.01) and 10% long-term survival (online supplemental figure 2D). Anatomopathological analyses of mouse brains sacrificed 15 days after viral inoculation revealed smaller or no tumors in both tumor models (online supplemental figure 2E). Additionally, analyses of the brains conducted 3 (CT-2A) and 6 (GL261-5) days after cell engraftment showed that incipient tumors could be observed even at these early time points (online supplemental figure 2F). In a second experiment, we compared whether treatment with Delta-24-ACT or Delta-24-RGD would produce differences in survival (figure 3A, B). We observed that both viruses increased the median survival of mice bearing CT-2A tumors compared with that of control mice (PBS=23 days, Delta-24-RGD=28.5 days and Delta-24-ACT=30 days; p=0.014 and p=0.002, respectively; figure 3C). Importantly, treatment with Delta-24-ACT also led to 35% long-term survival. In mice bearing GL261 tumors, although the difference was smaller, Delta-24-ACT showed superior efficacy than the control treatment (PBS=24 days, Delta-24-RGD=28 days and Delta-24-ACT=28.5 days; p=0.004; figure 3D) and led to 23% long-term survival.

10.1136/jitc-2021-002644.supp3 Supplementary data

Because the treatment of patients with agonistic anti-4-1BB antibodies has produced abnormalities in liver function, we next analyzed several hepatic parameters, including albumin, ALP, ALT, AST and BILT3. We did not find any significant difference in hepatic function between mice treated with Delta-24-ACT and those treated with PBS (figure 3E), which demonstrated that the intratumoral administration of the virus had no negative impact on the liver. Finally, we performed a rechallenge experiment to ascertain whether Delta-24-ACT-treated mice developed antiglioma immune memory. We observed that both naïve mice and long-term survivors died on similar days, which indicated an insufficient antiglioma immunological memory response (figure 3F).

### Delta-24-ACT recruits T lymphocytes to the tumor site and alters their immune phenotype

**Figure 4:**
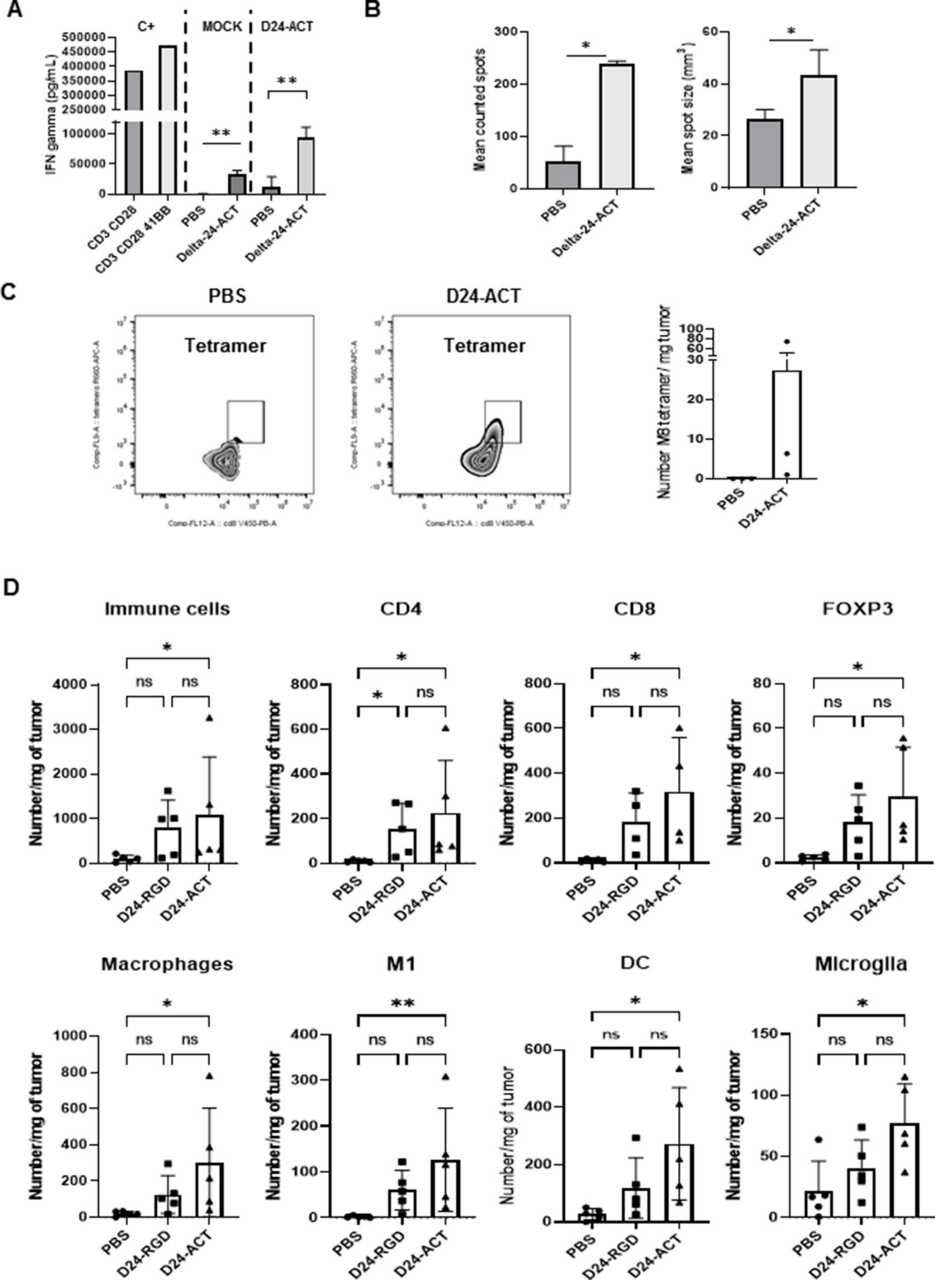
Modulation of the tumor immune microenvironment by Delta-24-ACT treatment. IFN-gamma secretion by splenocytes isolated from the spleen of mice treated with PBS or Delta-24-ACT and cocultured with mock- or Delta-24-ACT-infected CT-2A cells; the secretion was measured by ELISA (A). Assessment of mean counted spots (left panel, B) and mean spot size (right panel, B) obtained by ELISPOT of the coculture of splenocytes isolated from the spleen of mice treated with PBS or Delta-24-ACT with mock-infected CT-2A cells. (C) Analysis of tumor-infiltrating lymphocyte populations in the brains of mice bearing CT-2A tumors treated with Delta-24-ACT or PBS. The studied populations were CD45+ cells, CD4+ cells, CD8+ cells, CD4+ T regulatory (FOXP3+) cells, microglia, macrophages (F4/80+ cells), M1 cells and DCs. All markers were analyzed by flow cytometry (one-way ANOVA).

To evaluate whether the virus induces an antitumor immune response, we isolated splenocytes from control-treated and Delta-24-ACT-treated tumor-bearing mice and cocultured these cells with either mock-infected or infected cells for 3 days. IFN-gamma secretion was significantly increased in the supernatants of the cells cocultured with Delta-24-ACT-treated splenocytes compared with those of the cells cocultured with PBS-treated splenocytes (p=0.002 and p=0.013, respectively; figure 4A). These results demonstrated that Delta-24-ACT triggered an immune response not only against virus-specific antigens but also against tumors. Splenocytes incubated with anti-CD3 and anti-CD28 antibodies and splenocytes incubated with anti-CD3, anti-CD28 and anti-4-1BB antibodies were used as positive controls. In contrast, lymphocytes, non-infected cells and infected cells without coculture were used as negative controls (figure 4A). Additionally, we assessed the functionality of lymphocytes by IFN-gamma ELISPOT. Similarly, we observed significant increases in the number (PBS=52.33±29.27, Delta-24-ACT=239.33±5.18; p=0.012) and mean size (PBS=26.56 mm^3^±3.5, Delta-24-ACT=37.82 mm^3^±1.9; p=0.0063) of spots obtained from mock-infected cells cocultured with Delta-24-ACT-treated splenocytes compared with those obtained with PBS-treated splenocytes (figure 4B, left and right panels). These results suggested a specific antitumor response. In addition, the presence of MuLV p15E tetramer-positive CD8+ T cells in the tumors from mice treated with Delta-24-ACT demonstrated that the virus was able to induce an antitumor T cell response compared with PBS (figure 4C). In summary, these data support the therapeutic effect of Delta-24-ACT and suggest the triggering of an antitumor immune response.

To gain a deeper understanding of the mechanisms underlying the antitumor effect, we characterized the type and functional status of the immune populations that were recruited into tumors by Delta-24-ACT treatment. Tumor cells were implanted orthotopically, and 4 days later, the mice were randomized to the mock-treated, Delta-24-RGD-treated or Delta-24-ACT-treated groups. The mice were sacrificed 15 days later and processed for pathology or flow cytometry studies. Delta-24-ACT-treated brains displayed a significant increase in the accumulation of CD45+ cells compared with PBS-treated brains (p=0.01; figure 4D). Both viruses significantly increased CD4+ T cells (p=0.004 and p=0.01, respectively). Delta-24-ACT treatment increased the accumulation of CD8+ T cells (p*=*0.01) compared with that obtained with PBS treatment (figure 4D). Interestingly, we also observed a significant increase in the regulatory T cell (Treg) population (p=0.007; figure 4D), which suggested a potential mechanism of resistance. We did not observe differences in either NK or NKT cells between the control and virus-treated groups (online supplemental figure 3A). Because the brains were analyzed 15 days after treatment, we could not rule out the possibility that a first wave of NK cells accumulated very early during treatment but had already disappeared at the tested time point. Regarding myeloid cell populations, Delta-24-ACT treatment increased the numbers of microglia (p=0.03), macrophages (p=0.017) and DCs (p=0.01) compared with those obtained with PBS treatment. Furthermore, Delta-24-ACT increased the accumulation of proinflammatory macrophages (p=0.008, figure 4D). The analysis of myeloid-derived suppressor cell (MDSC) populations did not reveal significant differences in either the granulocyte-like or monocyte-like MDSC populations among the groups (online supplemental figure 3A).

10.1136/jitc-2021-002644.supp4 Supplementary data

Analyses of the functional status of these TILs showed that both the CD4+ and CD8+ cells in the Delta-24-ACT-treated mice displayed a significant increase in the level of the exhaustion marker PD-1 (p=0.01 and p=0.002, respectively; online supplemental figure 3B, C). Moreover, the CD4+ cell population in the Delta-24-ACT group displayed increases in the frequencies of GITR-positive (p=0.001) and granzyme B-positive cells compared with that in the PBS group (p=0.001; online supplemental figure 3B). The analysis of these markers in the CD8+ cell populations revealed no significant differences among the groups, although the frequencies of both markers tended to be higher in the treated group (online supplemental figure 3C). Analyses of the functional status of myeloid cells showed that macrophages (p<0.0001) from mice treated with both viruses displayed significant increases in PD-L1 expression compared with those from PBS-treated mice. In DCs, we also observed an increase in this marker with both virus treatments, although a greater increment was obtained with Delta-24-ACT (p=0.028 and p=0.007, respectively, online supplemental figure 4A). In addition, an increase in PD-L1 expression in microglia was only observed mice treated with Delta-24-ACT (p<0.027) compared with the control group (online supplemental figure 4A). Anatomopathological studies showed that mice bearing GL261-5 cells treated with Delta-24-ACT showed increased infiltration of CD3+ cells compared with PBS-treated mice (p=0.009; online supplemental figure 4B). Additionally, these studies showed that Delta-24-ACT did not exert any effect on angiogenesis, and no differences were detected in either the number or size of blood vessels between the Delta-24-ACT group and the PBS group (online supplemental figure 4C).

10.1136/jitc-2021-002644.supp5 Supplementary data

### The combination of Delta-24-ACT with a PD-L1 inhibitor alleviates CD8+ T cell exhaustion and induces a superior antiglioma immune response

**Figure 5:**
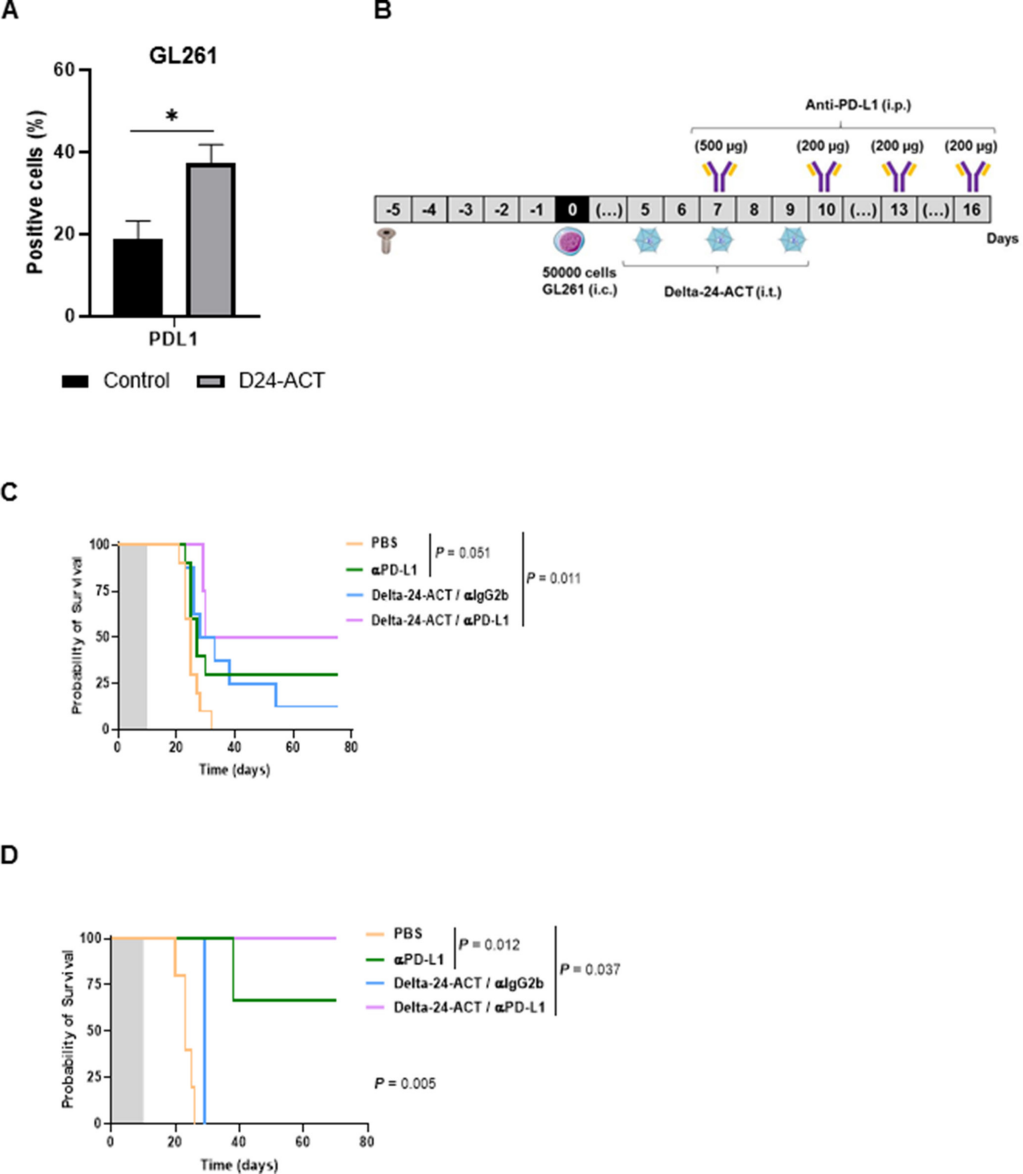
The combination of Delta-24-ACT and PD-L1 blockade in GL261 tumors increases survival and generates long-term protection. (A) GL261 cells were infected with Delta-24-ACT for 48 hours. The PD-L1 marker was analyzed by flow cytometry. (B) Schedule of the survival experiment with the GL261 tumor model. (C) Survival of mice bearing GL261 tumors. Mice were treated with an anti-PD-L1 antibody or Delta-24-ACT as a monotherapy or with the combination of Delta-24-ACT and the anti-PD-L1 antibody. (D) Rechallenge experiment performed with mice bearing GL261 tumors. GL261 cells were implanted in naïve mice or long-term survivors. All survival results are shown as Kaplan-Meier survival curves (log-rank test).

Because TILs from Delta-24-ACT-treated mice showed signs of exhaustion indicated by increased expression of PD-1, we next sought to evaluate whether the addition of an immune checkpoint inhibitor (ICI) would alleviate this exhaustion and result in an increased antitumor effect. In addition, since Delta-24-ACT significantly increased the expression of PD-L1 in GL261 cells in vitro compared with mock-treated cells (p=0.04, figure 5A) and because an increase in mice bearing GL261 tumors was observed in vivo (online supplemental figure 5A), targeting this immune checkpoint seemed to be an interesting approach. Therefore, we administered Delta-24-ACT according to a similar schedule as that described above with four intraperitoneal administrations of an anti-PD-L1 antibody (αPD-L1) to mice orthotopically injected with GL261 cells (figure 5B). The combination treatment resulted in a significant increase in the median survival of the treated animals (Delta-24-ACT/αPD-L1=52.5 days vs Delta-24-ACT/IgG2b=30.5 days, αPD-L1=27 days and PBS=25 days; p=0.011). More importantly, 50% of the animals administered the combination treatment shows long-term survival (figure 5C). Finally, we subjected the long-term survivors to rechallenge through the inoculation of the same cells to assess the potential generation of memory (figure 5D). All the PBS-treated mice died within 27 days. Importantly, 100% of the long-term survivors that were previously administered the combination treatment survived after rechallenge, which indicated that the combination treatment induced the development of antiglioma immune memory, whereas none of the animals administered Delta-24-ACT alone survived the rechallenge. Interestingly, 75% (two out of three) of the long-term survivors administered the anti-PD-L1 treatment were able to reject the rechallenge, which suggested that the blockade of this receptor might be important in the generation of immunological memory against the tumor in this model.

10.1136/jitc-2021-002644.supp6 Supplementary data

### The Delta-24-ACT/αPD-L1 combination remodels the tumor microenvironment leading to a proinflammatory myeloid and lymphoid population

**Figure 6:**
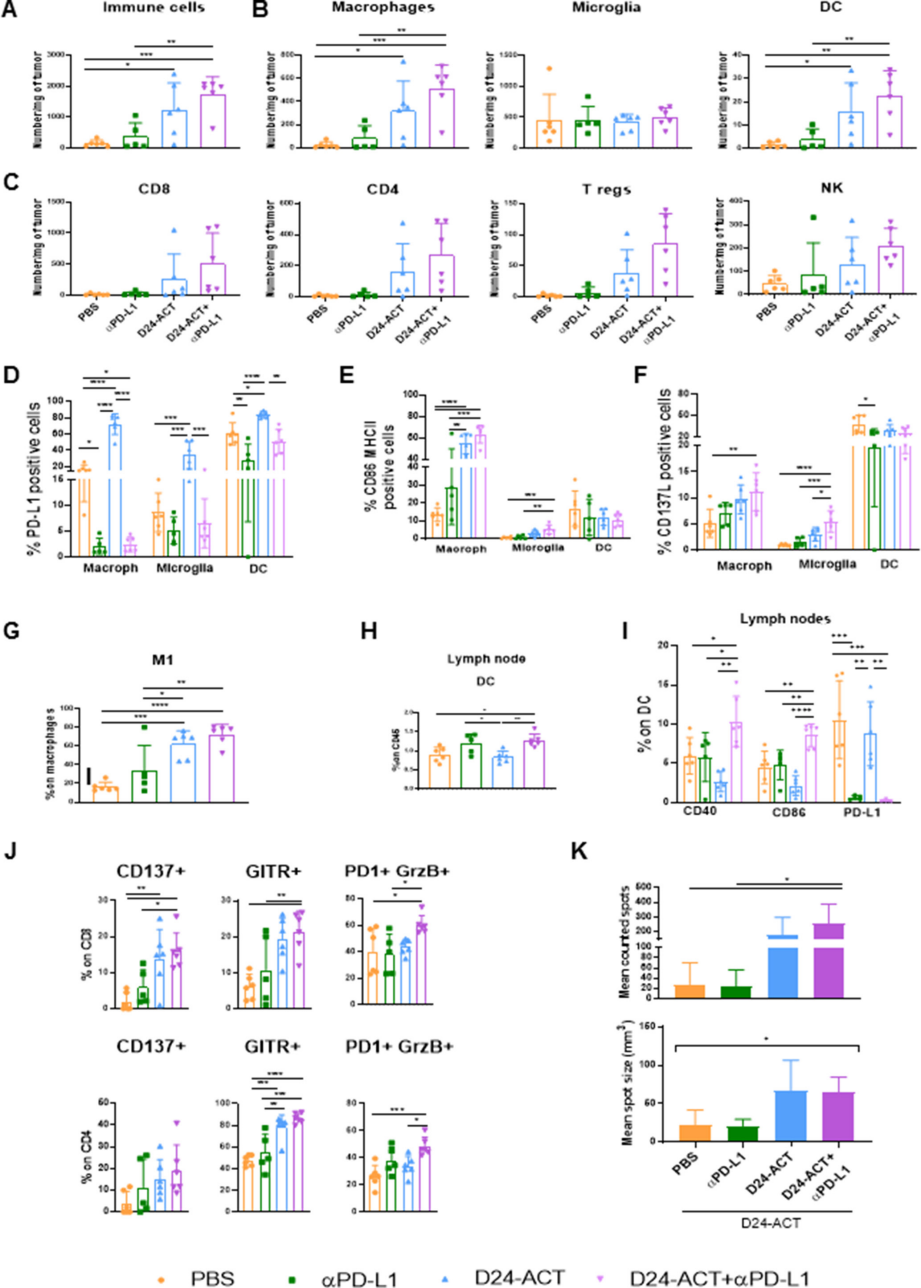
Modulation of the immune compartment in GL261 tumor-bearing mice after treatment with Delta-24-ACT in combination with PD-L1 antibody. Analysis of tumor-infiltrating lymphocyte populations in the brains of mice bearing GL261 tumors treated with PBS (control), αPD-L1, Delta-24-ACT or Delta-24-ACT/αPD-L1. The studied populations were CD45+ cells (A), myeloid cell populations, including macrophages, DCs and microglia (B), and lymphoid cell populations, including CD8+, CD4+, Tregs and NK cells (C). The analyses of the functional status of the myeloid cells described above included analyses of PD-L1 (D), CD86+MHCI+I (E), CD137L (F) markers and the percentage of M1 macrophages (G). Percentage of DCs present in the lymph nodes. (H) DC functional status. The CD40, CD86 and PD-L1 markers were analyzed (I). Phenotype of CD8+ and CD4+ T cells (J) based on studies of the CD137, GITR, PD-1 and GrzB markers. (K) Quantification of splenocyte IFN-gamma production by ELISPOT after the indicated treatment. Top panel: mean counted spots. Lower panel: mean spot size (one-way ANOVA).

Because the long-term survivors treated with the αPD-L1/Delta-24-ACT combination developed an immune memory response, we next studied the mechanisms involved. Mice-bearing GL261 cells were sacrificed 7 days after viral inoculation, and we analyzed the infiltrate in tumors and lymph nodes by flow cytometry. We observed a significant increase in the overall infiltration of immune cells (CD45+) in mice treated with either Delta-24-ACT alone or the combination compared with the mock-treated group (p=0.017 and p=0.0006, respectively, figure 6A gated in online supplemental figure 6B), which demonstrated that these treatments promote immune cell infiltration within the tumor. Furthermore, increased infiltration was observed in the combination group compared with the PD-L1 group (p=0.004, figure 6A). Interestingly, the combination treatment led to increases in the macrophage and DC populations when compared with those obtained with PBS (p=0.0007 and p=0.002, respectively) or PD-L1 (p=0.003 and p=0.009, respectively, figure 6B), which highlighted that the combination modulates the myeloid compartment. In contrast, no differences in microglia were detected among the groups (figure 6B). Moreover, within the lymphoid cell population, the combination treatment resulted in an increased tendency toward CD8, CD4, Tregs and NK cells (figure 6C). More importantly, analyses of the functional status of myeloid cells revealed that the Delta-24-ACT-treated mice showed higher PD-L1 expression than the control mice (p<0.0001, p=0.043 and p=0.0007). Moreover, reduced PD-L1 expression was detected in macrophages, microglia and DCs after PD-L1 monotherapy (p<0.0001, p=0.0003 and p<0.0001, respectively) or the combination (p<0.0001, p=0.0003 and p=0.003, respectively, figure 6D) compared with that observed in the control mice. These data were in accordance with the IHC findings showing that PD-L1 expression was reduced in tumors treated with αPD-L1 in combination with Delta-24-ACT (p=0.028, online supplemental figure 6A). The expression of the costimulatory molecule CD86 was higher in macrophages and microglia from tumors treated with Delta-24-ACT than in those from untreated mice. A greater increase in expression was obtained with the combination of Delta-24-ACT with PD-L1 blockade (p<0.0001 and p=0.0004, respectively, figure 6E), which highlighted that these cells were more active and had a proinflammatory phenotype. Finally, the expression of CD137L was higher in macrophages and microglia of the combination group than in those of the PBS group (p=0.008 and p<0.0001, respectively, figure 6F), which further supported the conclusion that these cells exhibit an active phenotype. We did not observe an increase in CD86 and CD137L markers in DCs (p=0.506 and p=0.17, figure 6E, F). Interestingly, the combination group showed a pronounced increase in M1 proinflammatory macrophages (gated as MHCII+CD11c+ from CD45hi/F4/80+ cells) (p<0.0001, figure 6G). Analyses of the lymph nodes revealed that the number of DCs in the combination group was higher than those in the PBS-treated and Delta-24-ACT-treated groups (p=0.017 and p=0.005, figure 6H). An evaluation of the expression of costimulatory molecules such as CD40 and CD86 in this population revealed an increase after the combination treatment (p=0.037 and p=0.002, respectively, figure 6I), which indicated activation and a more mature phenotype. In the lymph nodes, PD-L1 expression was lower in the groups treated with PD-L1 as monotherapy or in the combination treatment than in the PBS-treated group (p=0.0005 and p=0.0002, respectively, figure 6I). Tumor-infiltrating CD8+ cells showed a more active and cytotoxic phenotype in the combination group, as shown by the expression of the costimulatory molecules CD137 and GITR and the double-positive population PD-1+GrzB+ (p=0.001, p=0.002 and p=0.042, respectively, figure 6J). The tumor-infiltrating CD4+ cells in the combination group also showed higher expression of the costimulatory marker GITR and a greater proportion of PD-1+GrzB+ cells (p<0.0001 and p=0.0007, respectively, figure 6K). Finally, we evaluated the functionality of lymphocytes by IFN-gamma ELISPOT. Splenocytes from mice treated with the combination were more active than those from PBS-treated mice, as shown by increases in the number and mean size of the spots (p=0.02 and p=0.026, respectively, figure 6L). All these data suggest that Delta-24-ACT in combination with PD-L1 blockade promotes the infiltration of immune cells and alters their status within the tumor microenvironment to induce a proinflammatory microenvironment that allows induction of a better therapeutic effect.

10.1136/jitc-2021-002644.supp7 Supplementary data

## Discussion

Despite unparalleled preclinical and clinical research efforts, GBM remains a lethal tumor. The promising data derived from the use of ICIs in other cancers has prompted the evaluation of their efficacy in patients with GBM,^33^ but unfortunately, early trials showed no survival benefit in these patients.^34^ Pembrolizumab and nivolumab were well tolerated; however, progression-free survival and immune analyses have indicated that anti-PD-1 monotherapy is insufficient to produce an antitumor response in the majority of patients with GBM due to high levels of CD68+ cells and few T cells within the tumor microenvironment.^35 36^ The main limitations to implementing ICIs as a treatment for GBM include tumor localization; the presence of the blood-brain barrier, which makes it difficult for molecules to access tumors; and the global immune dysfunction state of patients, who have reduced lymphocyte populations as an effect of chemotherapy and radiotherapy.^37–39^ In this sense, OVs are interesting candidates for the intratumoral delivery of ICIs.

Targeting costimulatory members of the tumor necrosis factor receptor superfamily was found to be effective in the GL261 murine model, resulting in a 50% cure rate and increased median survival.^40 41^ Preclinical analyses conducted by other research groups have confirmed positive results employing OVs armed with other immunomodulators.^42 43^ Our results showed that the increase in the median survival of mice treated with Delta-24-ACT was associated with an increase in the infiltration of different immune cell populations, including cytotoxic T cells, into the tumor microenvironment. Surprisingly, myeloid populations remained unaltered after Delta-24-ACT administration. An engineered Vaccinia virus in combination with an anti-4-1BB antibody was also found to increase the accumulation of CD8+ T cells in a breast cancer model, but the study also showed increases in myeloid cell populations, particularly neutrophils.^44^ Some studies have demonstrated that the 4-1BB receptor is expressed in different immune cells, including T cells,^20 45 46^ and described that its interaction with 4-1BBL promotes the expansion of activated T cells and the generation and maintenance of memory CD8+ T cells.^47 48^ In our model, Delta-24-ACT as a monotherapy was not sufficient to cure all mice, and we could not ascertain the generation of immune memory. However, two recent studies using either an oncolytic adenovirus armed with 4-1BBL fused to the E7 antigen or a vaccinia Ankara virus armed with this same ligand showed the generation of immune memory in different tumor settings.^49 50^ One plausible explanation is that Delta-24-ACT replication is markedly attenuated in murine cells due to species-specific adenoviral replication.^51 52^ Therefore, in our model, we only observed a first wave of 4-1BBL expression, which might activate CD4+ and CD8+ T cells but be insufficient to sustain this activation or to costimulate new T cells that will be recruited into the tumor. Moreover, some studies have described viral instability associated with OVs armed with immunomodulators in the E3 region,^53^ resulting in inefficient antitumor activity. However, other researchers have reported that the increase in T cell accumulation elicited by an OV is associated with the induction of an antiglioma memory response, which has been shown to be specific for glioma antigens when inoculation with other nonglioma cells led to the development of tumors in mice.^16^ Interestingly, by evaluating different markers of activation and exhaustion in myeloid and lymphocyte populations, we noticed that both CD4+ T cells and CD8+ T cells in treated mice expressed higher levels of PD-1 than those in mock-treated mice, which suggested that the cells in treated mice exhibited a more exhausted phenotype. Moreover, several studies have demonstrated that the secretion of inflammatory cytokines induced by OVs upregulates PD-L1 in tumor cells, which is self-defeating for cytotoxic T cells.^54 55^ These findings suggest that the efficacy of Delta-24-ACT therapy could be improved using an anti-PD-L1 antibody,^56^ which could help alleviate the exhaustion induced by the activation of the immune response. In fact, evaluation of the combination of anti-PD-1 with Delta-24-RGD in the same glioma model showed superior efficacy than each single treatment.^57^ In our GL261 murine model, we did not observe changes in the immune phenotype in mice treated with αPD-L1 therapy alone compared with mice treated with PBS. Similar results were obtained in other studies administering both αPD-1 antibodies and αPD-L1 antibodies as single agents to syngeneic and genetically engineered GBM mouse models,^57–59^ which suggests the importance of combinatorial approaches. These data were consistent with other studies that employed different OVs in combination with ICIs, which achieved positive results.^60–62^ Other strategies combining an αPD-1 antibody with 4-1BB agonists have also shown increased therapeutic potential in subcutaneous CT26 colon carcinoma and B16F10 melanoma.^63 64^ Interestingly, these approaches have shown that PD-1 blockade increases 4-1BB expression in CD8+ T cells and that an anti-4-1BB antibody conversely induces PD-1 expression, which suggests a potential synergistic effect from the combination approach. Furthermore, dual therapy increases the CD8+ T cell/Treg ratio as well as the cytotoxic activity of T cells.^63^ However, the coadministration of anti-PD-1 and anti-4-1BB antibodies appears to heighten anti-4-1BB antibody-associated toxicities, which indicates the importance of selecting the correct administration method. In our models, the administration of Delta-24-ACT did not induce liver toxicity, which suggests that the intratumoral delivery of 4-1BBL circumvents the side effects reported by targeted antibodies due to the limited distribution achieved with this route. Moreover, the administration of an agonistic anti-4-1BB antibody as a monotherapy in murine glioma models led to an increase in median survival without effects on long-term survival. However, administration of the anti-4-1BB antibody in combination with PD-1 blockade not only resulted in a survival benefit but also yielded long-term survivors, decreased TIL exhaustion and improved TIL functionality.^65^ These results were in accordance with our results from the combination of Delta-24-ACT with PD-L1 blockade, which showed increases, although not significant, in the accumulation of CD4+ and CD8+ T cells and a reduction in proliferating cells in tumors treated with the combination compared with those treated with PBS (data not shown). Furthermore, we observed that the combination treatment induces changes in the status of lymphoid and myeloid cell populations exhibiting a proinflammatory phenotype.

We speculated that the immune memory response observed in mice treated with the combination was due to the influx of CD8+ T cells that did not undergo exhaustion therefore, these cells were able to become memory CD8+ T cells. A similar result was obtained with the administration of Delta-24-RGDOX, a virus that expresses OX40L, followed by anti-PD-L1 antibody in the GL261-5 murine model, which led to the development of immune memory that prevented tumor growth at a distant site.^15^

In summary, we proposed a combination-based strategy with OV Delta-24-ACT and an αPD-L1 antibody. This combinatorial approach increased T cell infiltration, reduced immunosuppression and led to an effective antitumor effect in murine glioma models. Moreover, our findings underscore the potential of combining local immunovirotherapy with ICI inhibitors as an effective therapy for poorly infiltrated tumors.

## Data Availability

The data that support the findings of this study are available within the paper or Supplemental Information or available from the corresponding author upon request.
