# Correction: *CD137 and PD-L1 targeting with immunovirotherapy induces a potent and durable antitumor immune response in glioblastoma models*

**DOI:** 10.1136/jitc-2021-002644corr1

**Published:** 2021-08-13

Puigdelloses M, Garcia-Moure M, Labiano S, *et al*. CD137 and PD-L1 targeting with immunovirotherapy induces a potent and durable antitumor immune response in glioblastoma models. *Journal for ImmunoTherapy of Cancer* 2021;9:e002644. doi: 10.1136/jitc-2021-002644

This paper has been updated since first published to amend author name 'Jaime Gállego Pérez-Larraya'.

