# Supplementary material for: CD137 and PD-L1 targeting with immunovirotherapy induces a potent and durable antitumor immune response in glioblastoma models
Source: J Immunother Cancer. 2021 Jul 19;9(7):e002644. doi: 10.1136/jitc-2021-002644 (PMC8291319; doi:10.1136/jitc-2021-002644)
Supplement: Supplementary data [file jitc-2021-002644supp001.pdf]

Puigdelloses et al.,

Supplementary Text and Figures

Supplementary figure 1. 4-1BBL expression in glioma cells after Delta-24-ACT infection. A. Quantification of E1A and Fiber bands from the WB in CT-2A and GL261-5 infected with Delta-24-ACT. **B.** E2F promoter activity in murine (CT-2A, GL261), human (U87-MG) glioma cell lines and NHA. **C.** Replication and quantification of wild type adenovirus (WT-Ad) and Delta-24-ACT in NHAs that were infected at an MOI of 10. **D.** Representative images showing the 4-1BBL expression in CT-2A cells infected with different Delta-24-ACT MOIs for 48 h. **E**. 4-1BBL protein expression in GL261 and U251-MG cells infected with Delta-24-ACT at the indicated MOIs analysed by western blot. A representative images are shown.

Supplementary figure 2. Comparison of the antitumor effect *in vivo* exerted by Delta-24-RGD and Delta-24-ACT in murine glioma models. Schedule of survival experiment in CT-2A (**A**) and GL261-5 (**B**) models. Survival of mice bearing CT-2A tumors (**C**) and GL261-5 tumors (**D**) treated with Delta-24-ACT ($1x10^8$ PFU/mouse, 3 µL, N = 10). A control group of mice treated with PBS was included in every survival experiment. **E.** H/E staining images of mice treated with Delta-24-ACT or PBS after being challenged with CT-2A (**C**) or GL261-5 (**D**) tumors. **F.** H/E staining images of mice engrafted with CT-2A and GL261-5 sacrificed the day of the virus inoculation. All survival experiments show the Kaplan-meier survival curves (Log-rank test).

Supplementary figure 3. Characterization of the tumor microenvironment in murine glioma tumors. A. Myeloid and lymphoid cell populations were analysed in brains of mice bearing CT-2A tumors treated with Delta-24-ACT, Delta-24-RGD or PBS. Analyzed populations were NK, NKT, LY6C and LY6G. All markers were evaluated by flow cytometry (One-way ANOVA). The functional status of CD4+ (**B**)

Puigdelloses M, *et al. J Immunother Cancer* 2021; 9:e002644. doi: 10.1136/jitc-2021-002644

and CD8+ (**C**) T cells was assessed by analyzing PD-1, GITR and GrzB. All markers were analyzed by flow cytometry (Student t-test).

Supplementary figure 4. Characterization and functional status of the tumor microenvironment in murine glioma tumors. A. PD-L1 expression in macrophages, DC and microglia from mice bearing CT-2A tumors treated with Delta-24-ACT, Delta-24-RGD or PBS. All markers were analysed by flow cytometry (One-way ANOVA). Anatomopathological analysis of CD3 (**B**) and CD31 (**C**) infiltration and quantification in mice bearing GL261-5 tumors treated with PBS or Delta-24-ACT analyzed by IHC (CD3) or IF (CD31) (Student t-test). Images were taken at 20x, scale bar 200 µm.

Supplementary figure 5. PD-L1 expression in glioma cells after Delta-24-ACT treatment. A. PD-L1 expression in in mice bearing GL261 tumors treated with PBS or Delta-24-ACT analysed by IHC. Images were taken at 20x. Scale bar 200 µm.

Supplementary figure 6. Analyses of PD-L1 expression after combination treatment. A. Quantification of PD-L1 expression in mice bearing GL261 tumors treated with the indicated treatments (PBS, αPD-L1, Delta-24-ACT / αIgG2b or Delta-24-ACT / αPD-L1 combination) by IHC (One-way ANOVA). Representative images are shown at 20x; scale bar 200 µm. **B.** Representative plots of the gating strategy of lymphoid and myeloid cell populations.
