# Supplementary figures and images for: CD137 and PD-L1 targeting with immunovirotherapy induces a potent and durable antitumor immune response in glioblastoma models
Source: J Immunother Cancer. 2021 Jul 19;9(7):e002644. doi: 10.1136/jitc-2021-002644 (PMC8291319; doi:10.1136/jitc-2021-002644)

## Supplementary Figure 1

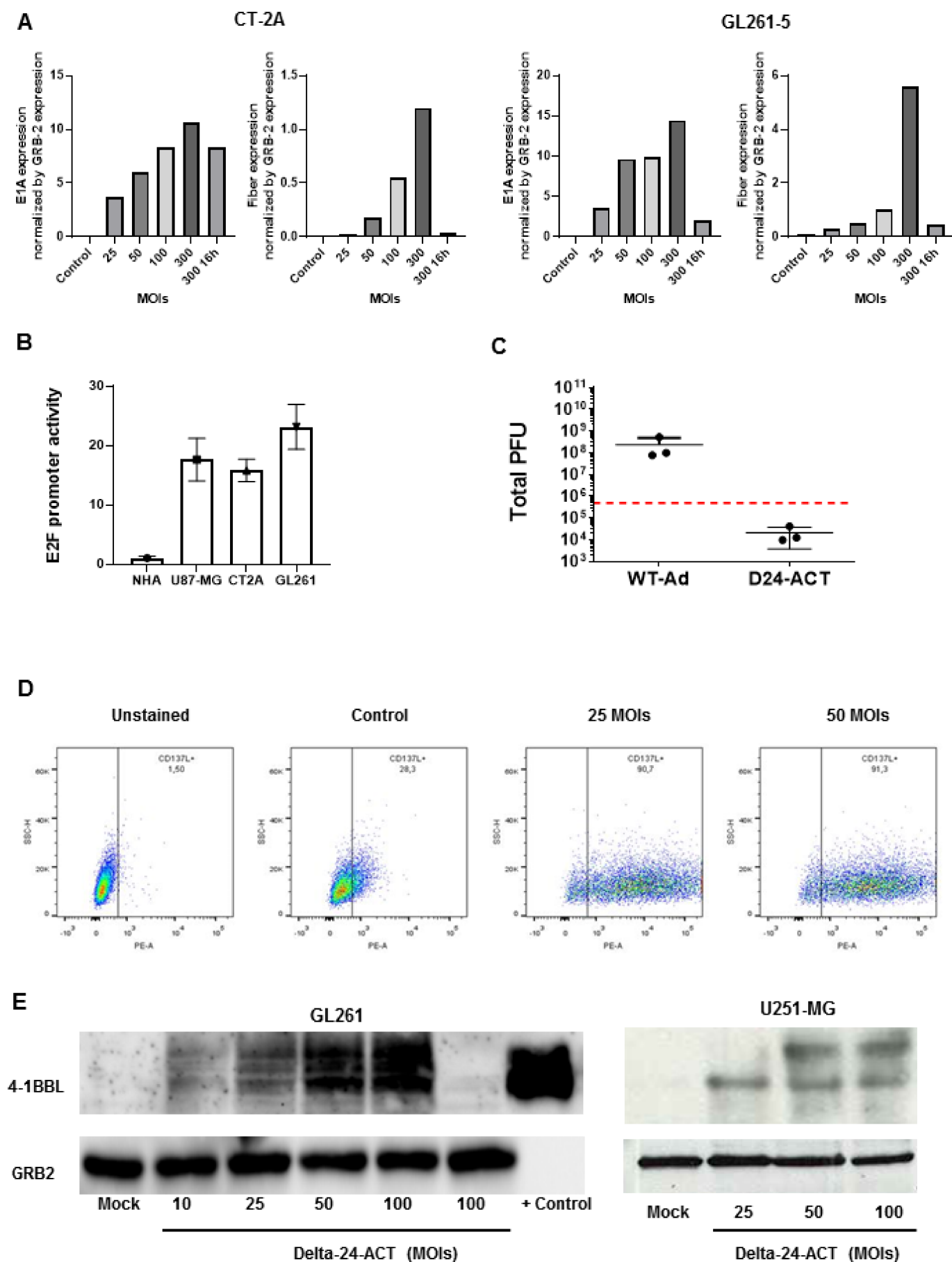

Supplement: Supplementary data [file jitc-2021-002644supp002.pdf]

## Supplementary Figure 2

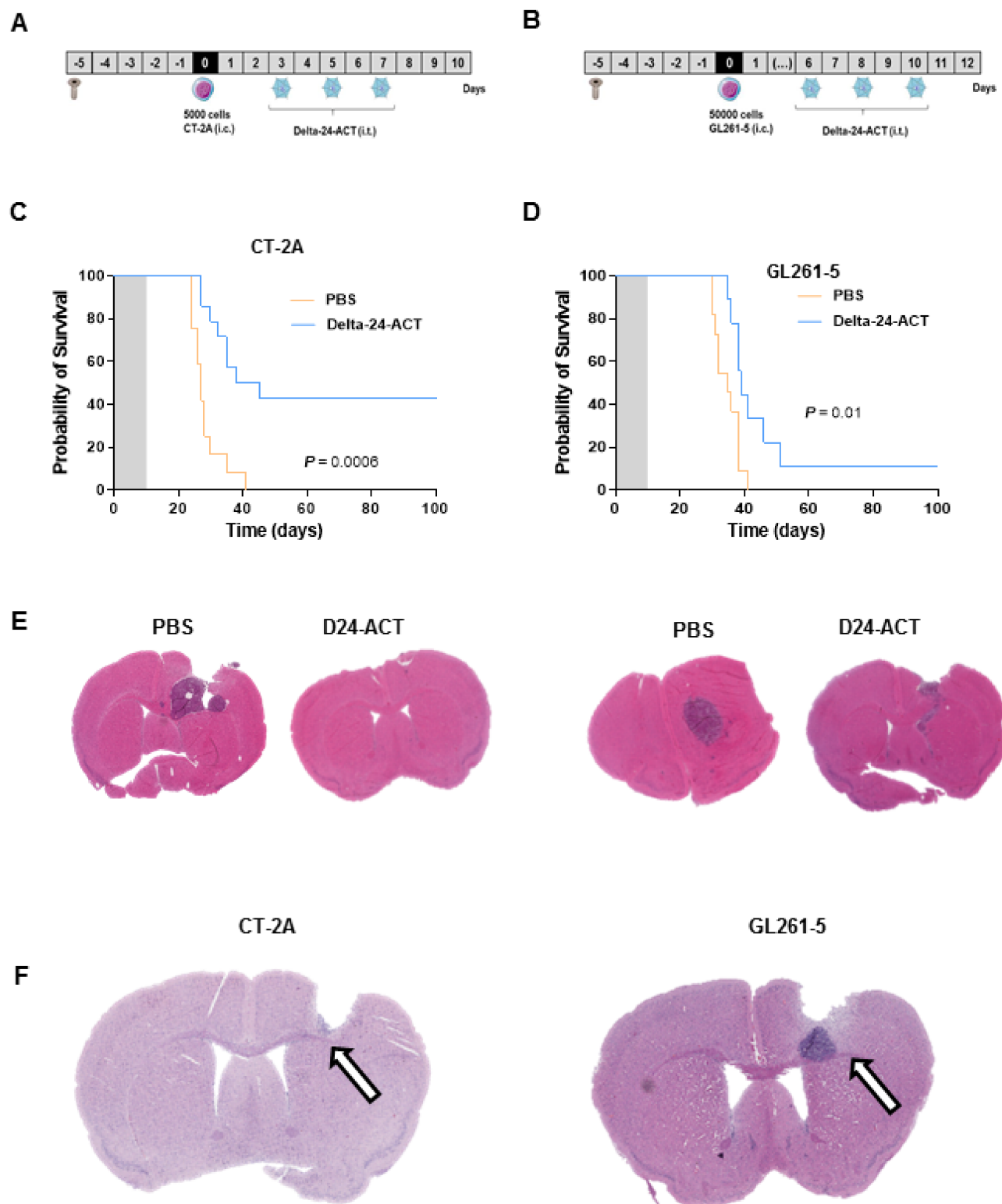

Supplement: Supplementary data [file jitc-2021-002644supp003.pdf]

A

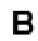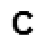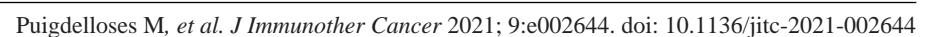

Supplement: Supplementary data [file jitc-2021-002644supp004.pdf]

## Supplementary Figure 4

A

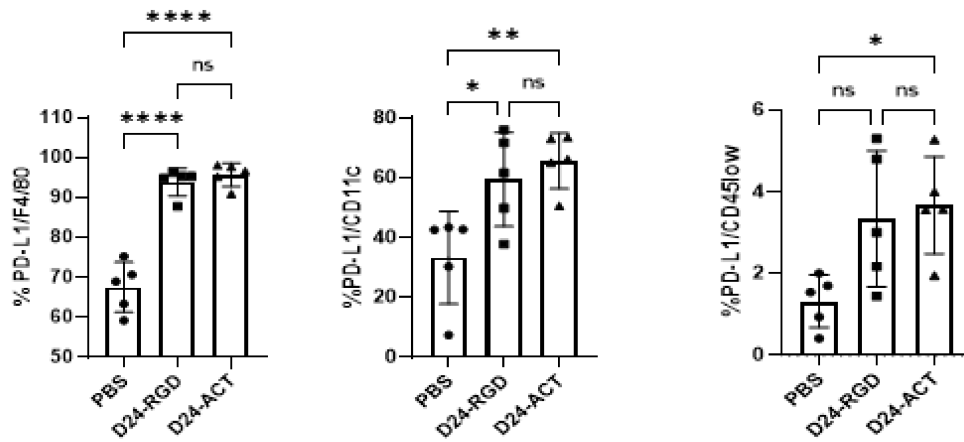

B

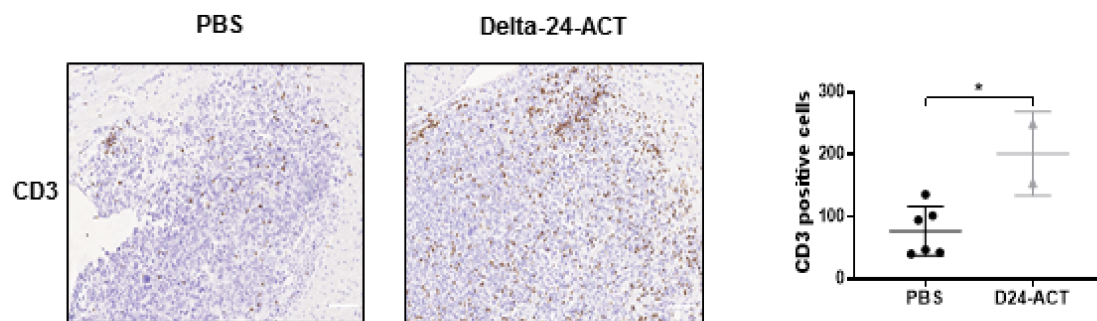

C

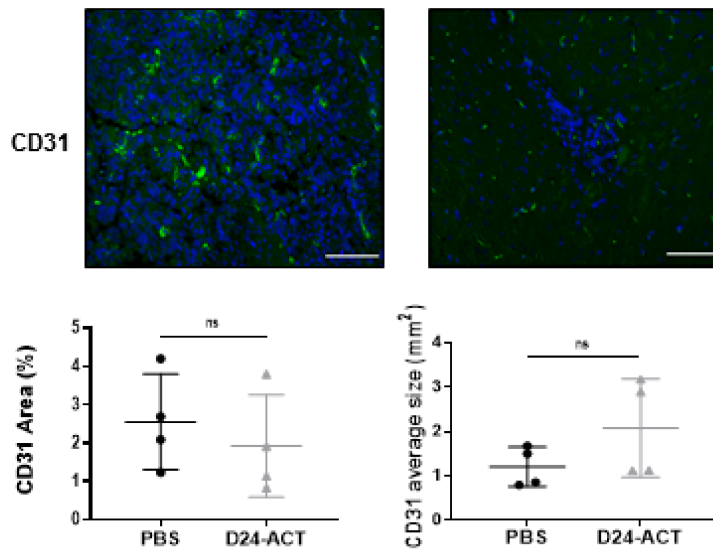

Supplement: Supplementary data [file jitc-2021-002644supp005.pdf]

**Supplementary Figure 5****A**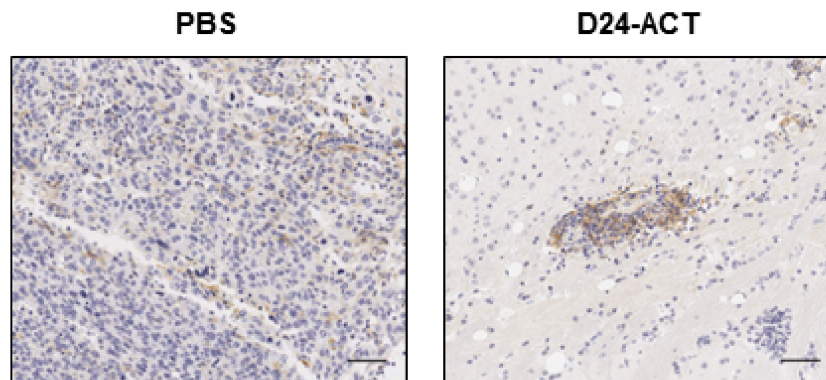

Supplement: Supplementary data [file jitc-2021-002644supp006.pdf]

**A**

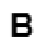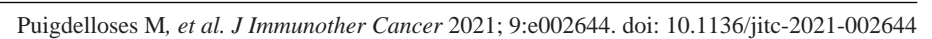

Supplement: Supplementary data [file jitc-2021-002644supp007.pdf]
